# Osteomalacia

**Published:** 1914-11

**Authors:** 


**Osteomalacia.** D. Cavarzani (Zentralb für Gyn.) reports another case treated with epinephrin, which now makes a total of forty-six cases. He believes the combination of hypophysis extract with the epinephrin treatment might enhance the value of the latter. Lime in the food has something to do.

